# SP1 and UTE1 Decoy ODNs inhibit activation and proliferation of hepatic stellate cells by targeting tissue inhibitors of metalloproteinase 1

**DOI:** 10.1186/s13578-016-0094-6

**Published:** 2016-05-10

**Authors:** Dong Jia, Yi-Ran Ni, Yan-Qiong Zhang, Chun Rao, Jun Hou, He-Qing Tang, Chang-Bai Liu, Jiang-Feng Wu

**Affiliations:** Medical College, China Three Gorges University, 8 Daxue Road, Xiling District, Yichang, 443002 Hubei Province China; Institute of Liver Diseases, China Three Gorges University, Yichang, Hubei Province China; Hubei Key Laboratory of Tumor Microenvironment and Immunotherapy, China Three Gorges University, Yichang, Hubei Province China; First Clinical Medical College, China Three Gorges University, Yichang, Hubei Province China

**Keywords:** SP1 Decoy ODN, UTE1 Decoy ODN, TIMP1, TGF-β signal pathway, Hepatic stellate cells

## Abstract

**Background:**

The excessive accumulation of extracellular matrix of hepatic fibrosis is positively correlated with tissue inhibitors of metalloproteinase 1 (TIMP1). Here we aimed to investigate whether TIMP1 may be down-regulated by Decoy ODNs strategy to capture transcriptional factor upstream TIMP1 element 1 (UTE1) and specificity protein 1(SP1).

**Results:**

By luciferase reporter assays, we confirmed that these Decoy ODNs could influence the promoter activation of *TIMP*-*1*, *α*-*SMA* and *Collagen Iα2* (*COLΙα2*) *genes* as well as the enhancer activation of TRE in HSC-T6 cells, and the combination tended to be more effective than SP1 or UTE1 Decoy ODN alone. Western blot analysis also demonstrated down-regulation of the expression of those target genes except for *TGF*-*β*. Furthermore, we observed that the viability of HSC-T6 cells at 72 h was significantly in decline in combination group.

**Conclusion:**

The combination of SP1 and UTE1 Decoy ODNs treatments inhibit the activation and proliferation of HSCs more effectively than one of the Decoy ODNs through co-regulation of TIMP1 and TGF-β signal pathway but not the expression of TGF-β itself.

## Background

Hepatic fibrosis is a common pathological progress into cirrhosis, which often leads to the death of the patient without liver transplantation, in a variety of liver diseases. A growing body of evidence indicates that hepatic fibrosis and even cirrhosis can be at least partially resolved when the disease cause is effectively prevented. However, the mechanisms of hepatic fibrosis and cirrhosis are still not completely understood, and there is no approved therapy yet to reverse this progression [1].

Hepatic fibrosis is characterized with the accumulation of extracellular matrix (ECM), including collagens, glycoproteins, and proteoglycans, and hepatic stellate cells (HSCs) are the major cells involved in ECM metabolism [2]. In normal liver, HSCs perform physiological function as the major hepatic store for retinoid in the space of Disse. In response to liver injury, HSCs go through progressive phenotypic transformation to proliferating myofibroblast-like cells [3] under stimulated with fibrotic cytokines and inflammatory cytokines, such as TGF-β, CTGF, PDGF [4, 5]. The activation of HSCs is characterized by loss of vitamin A droplets, expression of smooth muscle α-actin (α-SMA) [6] and generation a lot of ECM [7] for epithelial to mesenchymal transition (EMT) [8]. When engaged with high oxidized cholesterol level, the expression of the profibrogenic factor tissue inhibitors of metalloproteinase 1 (TIMP1), which prevents ECM being degraded by forming an inhibitory complex with the matrix metalloproteinases (MMPs) [9] and accelerates cell proliferation in a large-scale of cell types independent of its MMP-inhibitory activity [10], was also significantly up-regulated as well as TGF-β in HSCs of mice [11]. The expression of these hepatic fibrosis related genes are regulated by some transcription factors, such as specificity protein 1 (SP1), NF-κB, Smads, upstream TIMP1 element 1 (UTE1).

Decoy ODN is a short DNA segment from 10 to 30 bp including transcription factor binding site (TFBS) [12], which can competitively bind transcription factor and prevent the transcription factor binding to TFBS of target genes effectively,thus the Decoy ODNs strategy influences the expression of target genes through blocking mRNA transcription at the DNA level [13]. SP1 has highly homologous zinc-finger domains in the C-terminal region binding GC or GT boxes and glutamine-rich domains for transcriptional activation in the N-terminus. It regulates the expression of genes, including not only housekeeping genes, but also the tissue-specific gene [14]. SP1 is involved in the expression of ECM genes that have an important role in hepatic fibrosis progress and regulates expression of several genes that are relevant to downstream targets of TGF-β [15]. In addition, the overexpression of SP1 can also inhibit matrix metalloproteinase-9 (MMP-9) transcription, thus preventing matrix degradation [16]. SP1 Decoy ODN could inhibit the activation of HSCs [17]. UTE1 is an essential regulatory DNA motif for the activity of TIMP1 promoter in HSCs [18] and there is no report using UTE1 Decoy ODN to inhibit the activity of TIMP1 promoter yet.

There are GC-rich motifs binding for SP1 and TFBS for UTE1 on the TIMP1 promoter sequence inspiring us to explore whether combination of SP1 and UTE1 Decoy ODNs treatments can inhibit the activation of HSCs through capturing SP1 and UTE1 to down-regulate the activation of TIMP1. To test this hypothesis, we analyzed the influences of the combination of SP1 and UTE1 Decoy ODNs on the activation of promoter TIMP1 and the expression of TIMP1. Besides, we also analyzed the influences on TRE and the hepatic fibrosis related genes, *Collagen Iα2* (*COLΙα2)*, *α*-*SMA*, *TGF*-*β*, as well as the proliferation of HSC-T6 cells.

## Results

### The expression of TIMP1 was downregulated by SP1 and UTE1 Decoy ODNs treatment in HSC-T6 cells

**Fig. 1:**
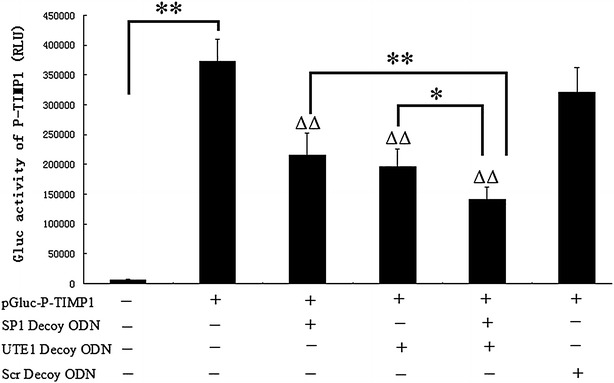
Influence of SP1 and UTE1 Decoy ODNs on the activity of promoter of TIMP1 in HSC-T6 cells. After pGLuc-P-TIMP1 was transfected into HSC-T6 cells for 24 h, Decoy ODNs were transfected for another 24 h. Data are presented as the mean ± SD of three experiments and each experiment for three wells. ^ΔΔ^p < 0.01 represented three experimental groups compared to Scr Decoy ODN group respectively. *p < 0.05 and **p < 0.01

There are three binding sites for transcription factor SP1 and one binding site for transcription factor UTE1 in the promoter of TIMP1 through bioinformatics analysis. To determine whether SP1 and UTE1 Decoy ODNs influence the activity of the promoter of TIMP1, we constructed plasmid pGLuc-P-TIMP1 which is Gaussia luciferase report gene for the promoter of TIMP1 and the results showed it was activated in HSC-T6 cells compared to mock (Fig. 1). After pGLuc-P-TIMP1 was transfected into HSC-T6 cells for 24 h, Decoy ODNs were transfected into HSC-T6 cells for another 24 h and the results showed all luciferase activities of the pGLuc-P-TIMP1 obviously decreased (p < 0.01) in three experimental groups (SP1 Decoy ODN group, UTE1 Decoy ODN group, mixture group of SP1 and UTE1 Decoy ODNs), respectively, compared to Scr Decoy ODN group. The results also showed the luciferase activities of the pGLuc-P-TIMP1 in mixture group of SP1 and UTE1 Decoy ODNs decreased compared with SP1 Decoy ODN group (p < 0.01) and UTE1 Decoy ODN group (p < 0.05), respectively (Fig. 1), suggesting the combination of SP1 and UTE1 Decoy ODNs can further inhibit the activation of promoter of TIMP1 than either of them.

**Fig. 2:**
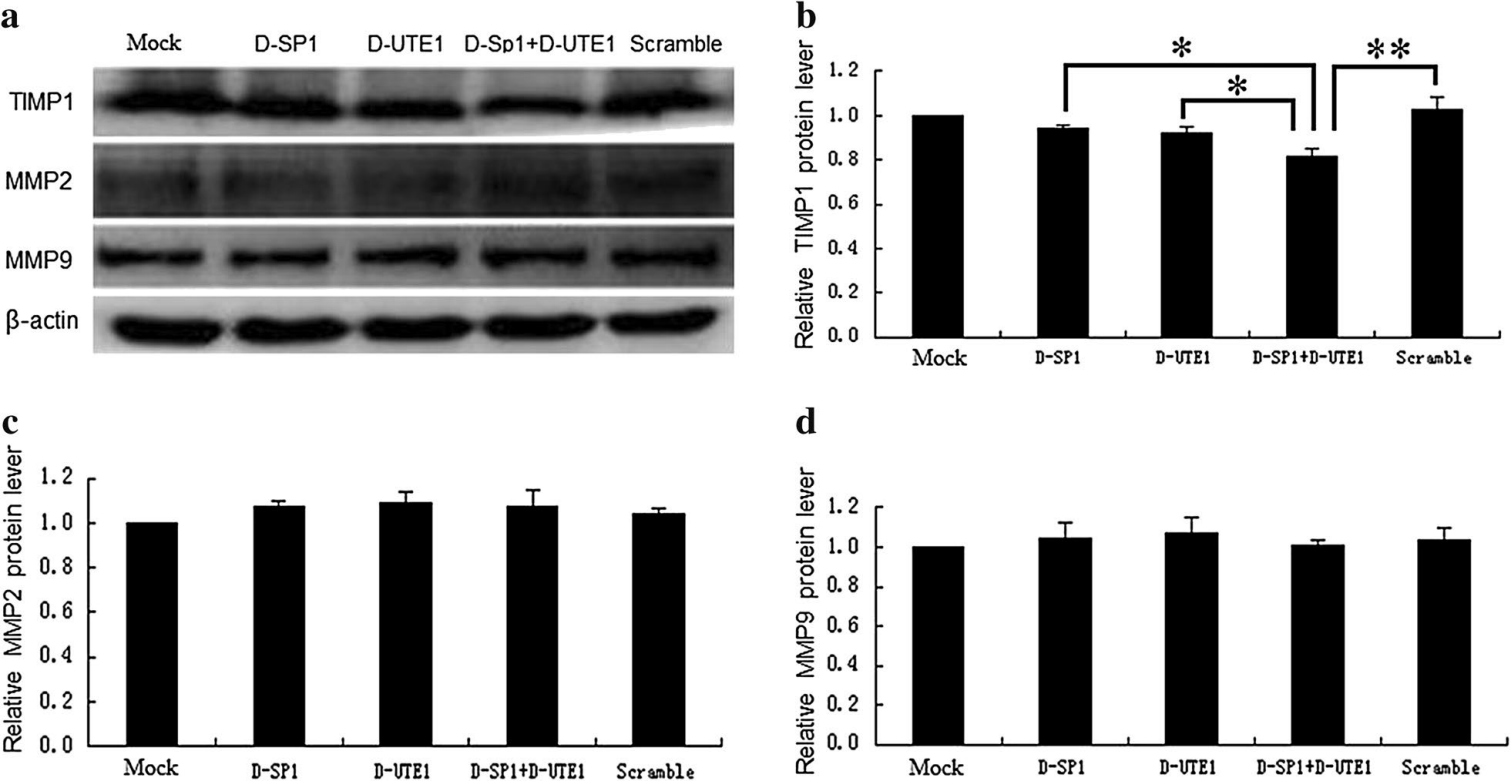
The expression of TIMP1, MMP2 and MMP9 dealed with SP1 and UTE1 Decoy ODNs by western blot assay. *D-SP1* SP1 Decoy ODN, *D-UTE1* UTE1 Decoy ODN, *Scramble* Scr Decoy ODN. Data are presented as the mean ± SD of three experiments. *p < 0.05 and **p < 0.01. The β-actin protein served as control and band intensities were normalized to β-actin in the quantificative analysis. **a** The expression of TIMP1, MMP2 and MMP9 were analysed by western blot assay when SP1 and UTE1 Decoy ODNs were transfected into HSC-T6 cells for 48 h. **b** Quantification of TIMP1 expression in HSC-T6 cells by western blot showed an obvious decrease (p = 0.001) in average band intensity with mixture group of SP1 and UTE1 Decoy ODNs, compared with scramble control, while quantification of TIMP1 expression did not show significant decreases comparing group of SP1 Decoy ODN (p = 0.153) or group of UTE1 Decoy ODN (p = 0.071) to scramble control, respectively. There are significant decreases comparing group of mixture group of SP1 and UTE1 Decoy ODNs with group of SP1 Decoy ODN (p = 0.026) and group of UTE1 Decoy ODN (p = 0.036), respectively. **c** Quantification of MMP2 expression in HSC-T6 cells by western blot did not show significant changes (p > 0.05) among them. **d** Quantification of MMP9 expression in HSC-T6 cells by western blot did not show significant changes (p > 0.05) among them

To further certify SP1 and UTE1 Decoy ODNs influence on the expression of the TIMP1 in the activated HSCs, we transfected Decoy ODNs into HSC-T6 cells for 48 h and tested the expression of the TIMP1 through quantification of western blot assays, no evident decrease was observed in SP1 Decoy ODN group (p > 0.05) or Decoy ODN UTE1 group (p > 0.05) compared to scramble control, respectively. Nevertheless, there was significant decrease in TIMP1 expression dealing with the combination of SP1 and UTE1 Decoy ODNs, not only compared to scramble control (p < 0.01), but also compared with SP1 Decoy ODN (p < 0.05) or UTE1 Decoy ODN groups (p < 0.05), respectively (Fig. 2a, b).

To explore whether the expression of MMP2 and MMP9 in activated HSCs is down-regulated by SP1 and UTE1 Decoy ODNs, Decoy ODNs were also transfected into HSC-T6 cells for 48 h again. Not only SP1 or UTE1 Decoy ODNs, but also combination of SP1 and UTE1 Decoy ODNs could not down-regulate the expression of MMP2 (p > 0.05) and MMP9 (p > 0.05) compared to scramble control (Fig. 2a, c, d) through quantification of western blot assays.

### SP1 and UTE1 Decoy ODNs treatment decreased COLΙα2 synthesis in HSC-T6 cells

**Fig. 3:**
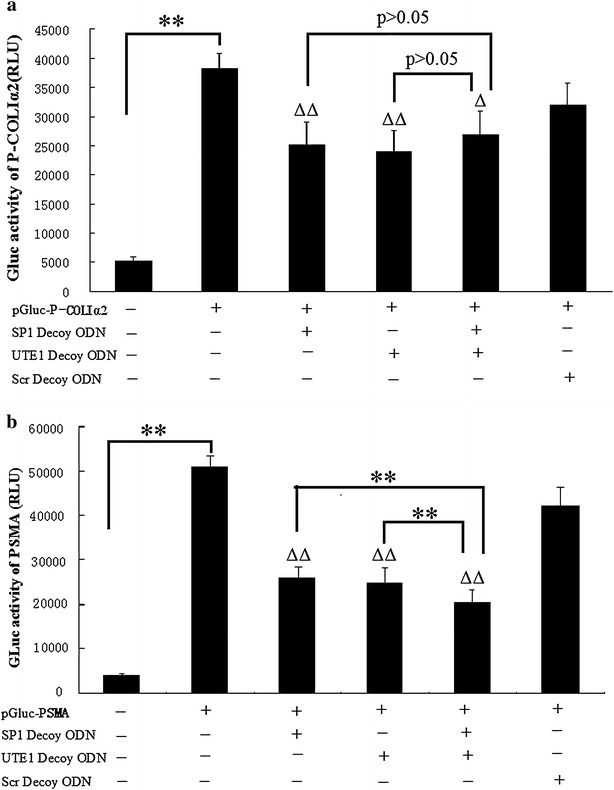
Influence of SP1 and UTE1 Decoy ODNs on the activity of promoters of COLIα2 and α-SMA in HSC-T6 cells. **a** After pGLuc-P-COLIα2 or **b** pGLuc-PSMA was transfected into HSC-T6 cells for 24 h, Decoy ODNs were transfected for another 24 h. Data are presented as the mean ± SD of three experiments and each experiment for three wells. ^ΔΔ^p < 0.01 and ^Δ^p < 0.05 three experimental groups compared to Scr Decoy ODN group respectively. **p < 0.01

Bioinformatics analysis found that there are two binding sites for transcription factor SP1 and no binding site for transcription factor UTE1 in the promoter of COLΙα2. To explore the influence on the activity of promoter of COLΙα2 by SP1 and UTE1 Decoy ODNs, the Gaussia luciferase report gene plasmid pGLuc-P-COLΙα2 for the promoter of COLΙα2 was constructed and the results showed it was activated in HSC-T6 cells comparing with mock (Fig. 3a). After pGLuc-P-COLΙα2 was transfected into HSC-T6 cells for 24 h, Decoy ODNs were transfected into HSC-T6 cells for another 24 h and the results showed all luciferase activities of the pGLuc-P-COLΙα2 evidently decreased (p < 0.01; p < 0.01; p < 0.05) in three experimental groups (SP1 Decoy ODN group, UTE1 Decoy ODN group, mixture group of SP1 and UTE1 Decoy ODNs), respectively, compared to Scr Decoy ODN group. However, there was no obvious difference in the luciferase activities of the pGLuc-P-COLΙα2 in mixture group of SP1 and UTE1 Decoy ODNs compared with SP1 Decoy ODN group (p > 0.05) or UTE1 Decoy ODN group (p > 0.05), respectively (Fig. 3a).

**Fig. 4:**
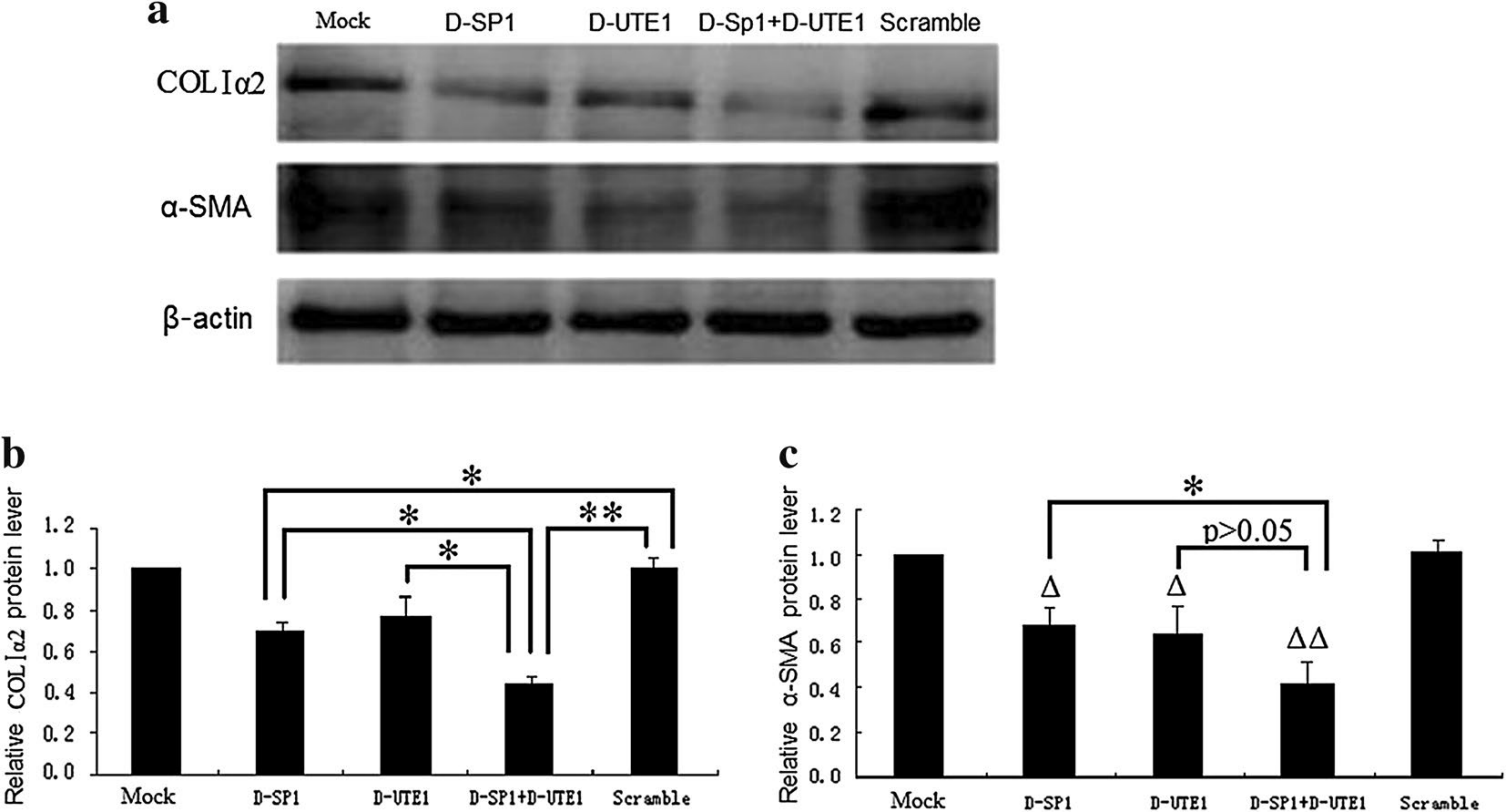
The expression of COLIα2 and α-SMA dealed with SP1 and UTE1 Decoy ODNs by Western blot assay. *D-SP1* SP1 Decoy ODN, *D-UTE1* UTE1 Decoy ODN, *Scramble* Scr Decoy ODN. Data are presented as the mean ± SD of three experiments. ^Δ^p < 0.05 and ^ΔΔ^p < 0.01 represented three experimental groups compared to Scr Decoy ODN group respectively. *p < 0.05 and **p < 0.01. The β-actin protein served as control and band intensities were normalized to β-actin in the quantificative analysis. **a** The expression of COLIα2 and α-SMA was analysed by western blot assays when SP1 and UTE1 Decoy ODNs were transfected into HSC-T6 cells for 48 h. **b** Quantification of COLIα2 expression in HSC-T6 cells by western blot showed a significant decrease (p = 0.034) in average band intensity with group of SP1 Decoy ODN, however, there was not obvious difference from group of UTE1 Decoy ODN (p = 0.220), compared to scramble control, respectively. There are significant decreases comparing group of combinational treatment of SP1 and UTE1 Decoy ODNs, not only to scramble control (p = 0.001), but also with group of SP1 Decoy ODN (p = 0.044) or group of UTE1 Decoy ODN (p = 0.030), respectively. **c** Quantification of α-SMA expression in HSC-T6 cells by western blot showed obvious decreases (p = 0.020; p = 0.010; p = 0.000) in average band intensity with three experimental groups (SP1 Decoy ODN group, UTE1 Decoy ODN group, mixture group of SP1 and UTE1 Decoy ODNs) compared with scramble control, respectively. There was a significant decrease (p = 0.045) comparing mixture group of SP1 and UTE1 Decoy ODNs with SP1 Decoy ODN group, however, there was no obvious difference (p = 0.179) compared to UTE1 Decoy ODN group, respectively

To further certify whether SP1 and UTE1 Decoy ODNs can down-regulate the expression of COLΙα2 in activated HSCs, Decoy ODNs were also transfected into HSC-T6 cells for 48 h. The results were analyzed by western blot and showed the expression of the COLΙα2 significant decrease (p < 0.05) dealing with SP1 Decoy ODN. However, there was no obvious difference (p > 0.05) between UTE1 Decoy ODN and scramble control. Moreover, there was significant decrease in COLΙα2 expression dealing with combination of SP1 and UTE1 Decoy ODNs, not only compared to scramble control (p < 0.01), but also compared with SP1 Decoy ODN (p < 0.05) or UTE1 Decoy ODN groups (p < 0.05), respectively (Fig. 4a, b).

### α-SMA expression was downregulated by SP1 and UTE1 Decoy ODNs in HSC-T6 cells

α-SMA is an important marker of activated HSCs. To explore the influence of SP1 and UTE1 Decoy ODNs on the activity of promoter of α-SMA, we found four binding sites for transcription factor SP1 and no binding site for transcription factor UTE1 in the promoter of α-SMA through bioinformatics analysis. When plasmid pGLuc-PSMA which is Gaussia Luciferase report gene for the promoter of α-SMA was transfected into HSC-T6 cells, the luciferase activity increased comparing with mock (Fig. 3b). After pGLuc-PSMA was transfected into HSC-T6 cells for 24 h, Decoy ODNs were transfected into HSC-T6 cells for another 24 h and the results showed all luciferase activities of the pGLuc-PSMA obviously decreased (p < 0.01) in three experimental groups (SP1 Decoy ODN group, UTE1 Decoy ODN group, mixture group of SP1 and UTE1 Decoy ODNs), respectively, compared to Scr Decoy ODN group. There were obvious decreases in the luciferase activities of the pGLuc-PSMA in mixture group of SP1 and UTE1 Decoy ODNs compared to SP1 Decoy ODN group (p < 0.01) or UTE1 Decoy ODN group (p < 0.01), respectively (Fig. 3b).

To further certify whether SP1 and UTE1 Decoy ODNs can down-regulate the expression of α-SMA in activated HSCs, Decoy ODNs were also transfected into HSC-T6 cells for 48 h. The results were analyzed by western blot and showed the expression of the α-SMA significant decrease dealing with only SP1 Decoy ODN (p < 0.05) or UTE1 Decoy ODN (p < 0.05), compared to scramble control, respectively. Notably, the expression sharply decreases, dealing with the combination of SP1 and UTE1 Decoy ODNs, not only compared to scramble control (p < 0.01), but also compared to SP1 Decoy ODN (p < 0.05), respectively. However, there was no obvious difference (p > 0.05) between mixture group of SP1 and UTE1 Decoy ODNs and group of UTE1 Decoy ODN (Fig. 4a, c).

### SP1 and UTE1 Decoy ODNs had no affect on TGF-β expression, but suppressed TRE activity partly in HSC-T6 cells

**Fig. 5:**
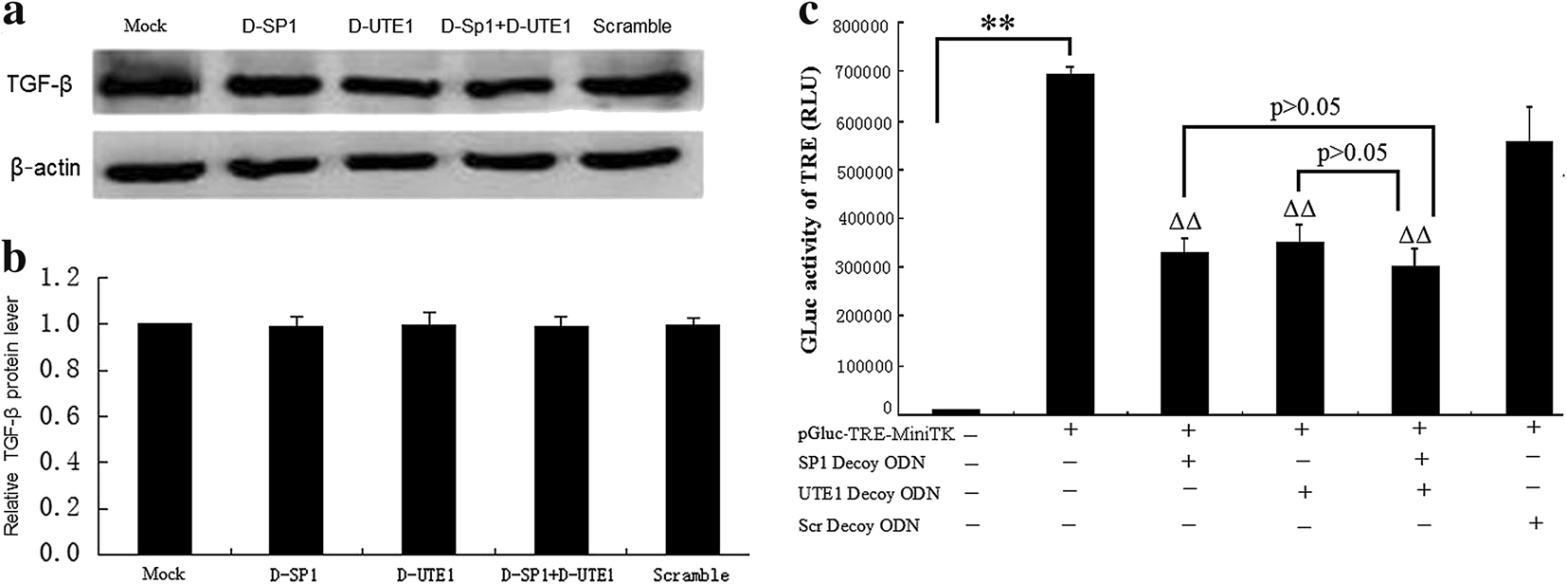
Influnce of SP1 and UTE1 Decoy ODNs on TGF-β and TRE in HSC-T6 cells. **a** Influnce of SP1 and UTE1 Decoy ODNs on the expression of TGF-β by western blot assays in HSC-T6 cells. The β-actin protein served as control. *D-SP1* SP1 Decoy ODN, *D-UTE1* UTE1 Decoy ODN, *Scramble* Scr Decoy ODN. **b** Quantification of TGF-β expression in HSC-T6 cells by western blot did not show significant changes (p > 0.05) among the groups. Data are presented as the mean ± SD of three experiments. The band intensities were normalized to β-actin in the quantificative analysis. **c** Influence of SP1 and UTE1 Decoy ODNs on the activity of TRE in HSC-T6 cells. After pGLuc-TRE-MiniTK was transfected into HSC-T6 cells for 24 h, Decoy ODNs were transfected for another 24 h. Data are presented as the mean ± SD of three experiments and each experiment for three wells. ^ΔΔ^p < 0.01 represented three experimental groups compared to Scr Decoy ODN group respectively. **p < 0.01

There are thirteen binding sites for transcription factor SP1 and no binding site for transcription factor UTE1 in the promoter of TGF-β through bioinformatics analysis. To explore the influence of SP1 and UTE1 Decoy ODNs on the expression of TGF-β, Decoy ODNs were also transfected into HSC-T6 cells for 48 h. The results were analyzed by western blot and showed the expression of the TGF-β was not influenced, not only in the group of SP1 Decoy ODN (p > 0.05) or group of UTE1 Decoy ODN (p > 0.05), but also in the mixture group of SP1 and UTE1 Decoy ODNs (p > 0.05), compared to scramble control, respectively. In addition, there was no obvious difference between mixture group of SP1 and UTE1 Decoy ODNs and group of SP1 Decoy ODN (p > 0.05) or group of UTE1 Decoy ODN (p > 0.05), respectively (Fig. 5a, b).

To explore the influence of SP1 and UTE1 Decoy ODNs on TRE, plasmid pGLuc-TRE-MiniTK which is Gaussia Luciferase report gene for the TRE was transfected into HSC-T6 cells and the result showed it was activated in HSC-T6 cells (Fig. 5c). After pGLuc-TRE-MiniTK was transfected into HSC-T6 cells for 24 h, Decoy ODNs were transfected into HSC-T6 cells for another 24 h and the results showed all luciferase activities of the pGLuc-TRE-MiniTK decreased obviously (p < 0.01) in three experimental groups (SP1 Decoy ODN group, UTE1 Decoy ODN group, mixture group of SP1 and UTE1 Decoy ODNs) compared to Scr Decoy ODN group, respectively. However, no evidence supported the luciferase activity of the pGLuc-TRE-MiniTK in mixture group of SP1 and UTE1 Decoy ODNs had any distinction compared with SP1 Decoy ODN group (p > 0.05) and UTE1 Decoy ODN group (p > 0.05), respectively (Fig. 5c).

### Combination SP1 and UTE1 Decoy ODNs treatment inhibited proliferation of HSC-T6 cells

**Fig. 6:**
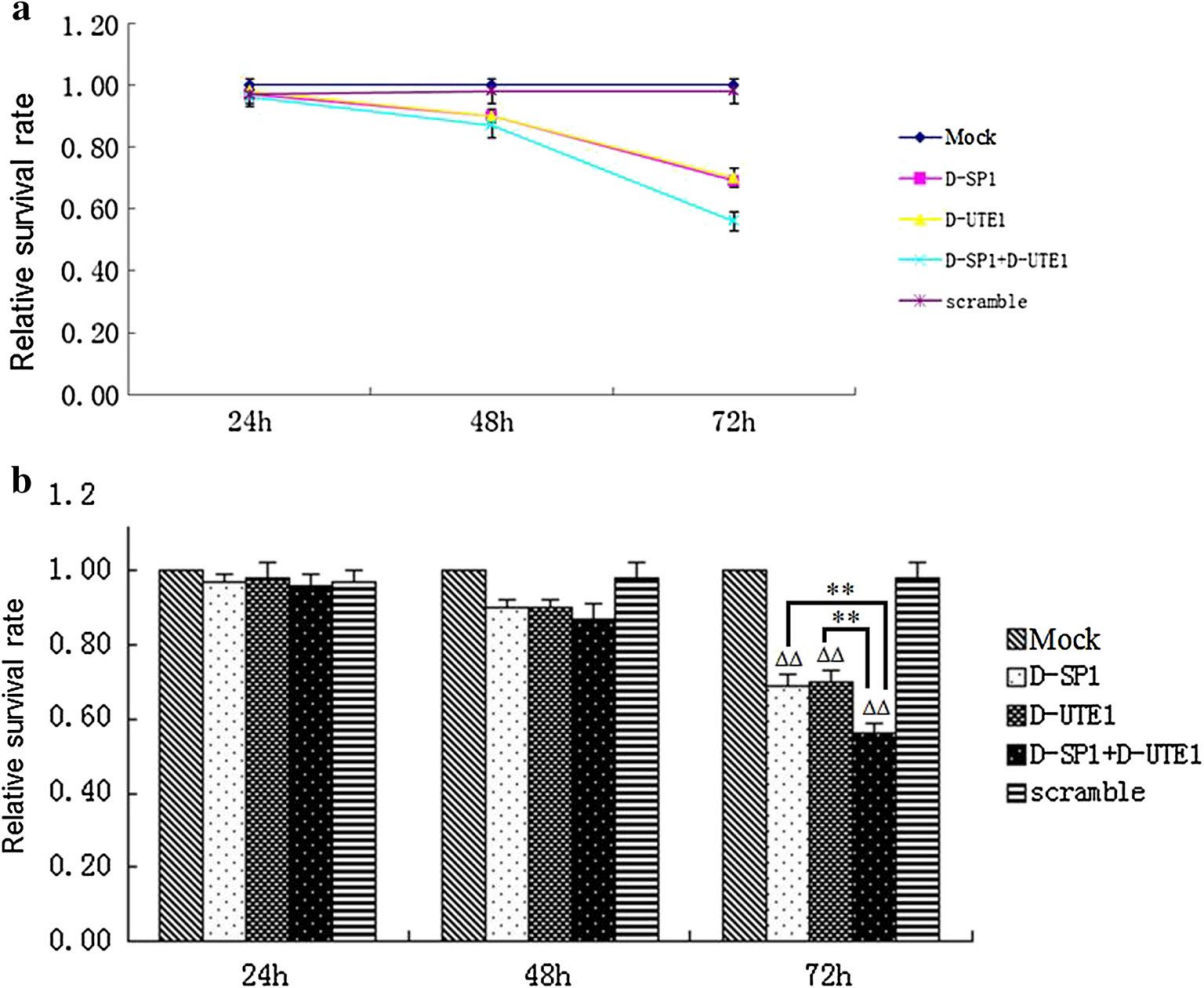
Inhibitory effect of SP1 and UTE1 Decoy ODNs on the proliferation of HSC-T6 cells. **a** After SP1 and UTE1 Decoy ODNs were transfected into HSC-T6 cells at 24, 48 and 72 h, the proliferation of cells were checked by MTT. **b** After Decoy ODNs transfected into HSC-T6 cells for 72 h, the survival rate obviously declined (p < 0.01) in SP1 Decoy ODN group and UTE1 Decoy ODN group at 72 h, compared to Scr Decoy ODN group. Data are presented as the mean ± SD of three experiments and each experiment for three wells. ^ΔΔ^p < 0.01 represented three experimental groups compared to Scr Decoy ODN group respectively. **p < 0.01

To explore whether SP1 and UTE1 Decoy ODNs can inhibit the proliferation of HSC-T6 cells, Decoy ODNs were transfected into HSC-T6 cells for 24, 48 and 72 h and the proliferation of HSC-T6 cells were checked by MTT assay. The results showed that all experimental groups had no obvious effect on the proliferation of HSC-T6 cells at 24 or 48 h. However, the survival rate obviously declined (p < 0.01) in SP1 Decoy ODN group and UTE1 Decoy ODN group at 72 h, compared to Scr Decoy ODN group, respectively. After SP1 and UTE1 Decoy ODNs were delivered together into HSC-T6 cells for 72 h, we found the cell survival rate was below 0.6, obviously declined compared with SP1 Decoy ODN group (p < 0.01) or UTE1 Decoy ODN group (p < 0.01), respectively (Fig. 6).

## Discussion/conclusion

HSCs play an important role in the progression of liver fibrosis when they activated and differentiated into myofibroblast cells which can synthesize ECM along with the expression of marker molecule α-SMA [19]. It is well known that over deposition of ECM can impair the liver function and collagen is the main component of ECM produced by activited HSCs [20]. In activated HSCs, down-regulation of SP1 is able to inhibit the proliferation and the expression of TGF-β, α-SMA and Smad4 [21]. In human hypertrophic scar fibroblasts, SP1 Decoy ODN can significantly reduce the secretion of collagen [22]. Blocking the effect of SP1 by SP1 Decoy ODN inhibits COLIα2 promoter activity in NIH 3T3 fibroblasts [23]. SP1 Decoy ODN can inhibit the activation of HSCs via down-regulating the expression of hepatic fibrosis related genes, such as *TGF*-*β*, *PDGF*-*BB*, *α*-*SMA*, *Collagen Iα1 (COL Iα1)* and *TIMP1* [17]. Our study indicated the activity of promoter of *α*-*SMA* and *COLIα2* genes and these genes expression can be inhibited by SP1 or UTE1 Decoy ODNs or their combination. In renal tubulointerstitial fibrosis, Smad/SP1 chimeric Decoy ODN have a more significantly inhibitory effect on EMT and fibrosis compared with Smad or SP1 Decoy ODNs [24]. In our study, we also proved it was more efficient for the combination of SP1 and UTE1 Decoy ODNs in inhibiting the activity of promoter of *α*-*SMA* gene and the expression of α-SMA and COLIα2 than one of Decoy ODNs. We cannot ignore the fact that GC-rich motifs exist in the promoters of *α*-*SMA* and *COLIα2* genes, but none binding sites for transcription factor UTE1 by bioinformatics analysis. However, we found the expression of *α*-*SMA* and *COLIα2* genes was further down-regulated by the combination of SP1 and UTE1 Decoy ODNs. The paradox requires further study. The excessive accumulation of ECM is derived from the imbalance of its synthesis and degradation [25]. The activation of TGF-β signal pathway, always mentioned for its association with ECM synthesis, regulates the expression of its target genes, such as collagen I, α-SMA and TIMP1. On the other hand, TIMP1 forms inhibitory complex with MMPs to prevent ECM from being degraded by active forms of MMPs [9].

ECM is mainly degraded by MMPs, including MMP2 and MMP9 [26] and this process can be inhibited by TIMP1 [27]. TIMP1 transgenic mice hardly engage hepatic fibrosis without any treatment, however, hepatic fibrosis can be induced easily dealing with CCl_4_ and an active form of MMP2 level in the liver decreases. Thus, TIMP-1 can strongly promote hepatic fibrosis development through forming an inhibitory complex with MMP2 [28]. Latest research found TIMP1 significantly enhanced expression of TGFβ1, α-SMA, collagen I and induced transformation of fibroblasts into myofibroblasts in urethral scar [29]. In the promoter of *TIMP1* gene, there presents a regulatory element (5′-TGTGGTTTCCG-3′) which can be bound by transcription factor UTE1 [30] and three GC-rich motifs for SP1. The activity of these regulatory elements in promoter of *TIMP1* gene could be inhibited effectively by UTE1 Decoy ODN or SP1 Decoy ODN in our experiment. Although the expression of TIMP1 did not obviously change dealing with single Decoy ODN, it could be down-regulated effectively by the combination of SP1 and UTE1 Decoy ODNs. In addition, MMP2 and MMP9 were not affected by single or combination of Decoy ODNs. Taken together, we discover that the combination of SP1 and UTE1 Decoy ODNs can effectively down-regulate the expression of COLIα2 and α-SMA, and the reasonable explanation is the combination can increase active forms of MMPs by inhibiting the activity of TIMP1.

TGF-β, a central regulator in chronic liver disease, contributes to fibrogenesis through inflammation and acts as an autocrine anabatic regulator for ECM production in the activated HSCs [31]. In HCV infection, TGF-β promoter activation is regulated by transcription factors SP1, AP-1, STAT-3, and NF-kB and the activation of these transcription factors is related to the activation of cellular kinases [32]. TGF-β can promote the expression of its target genes, α-SMA and collagen I, through Smad2/3/4 complex binding to TRE which is an enhancer of TGF-β target genes. Although SP1 and UTE1 Decoy ODNs could not down-regulate the expression of TGF-β, we found they could influence the activation of TRE. Furthermore, we observed that the combination of SP1 and UTE1 Decoy ODNs had a more powerful influence on the expression of α-SMA and COLIα2, but no different influence on the activation of TRE compared with single ODN. Thus, it may be part of the causes for the down-regulations of the expression of α-SMA and COLIα2 through TGF-β signal pathway being acted by the combination of SP1 and UTE1 Decoy ODNs. It also suggests that the combination of SP1 and UTE1 Decoy ODNs correct the inbalance of α-SMA and ECM from synthesis and degradation through TIMP1 and TGF-β signal pathway but not the expression of TGF-β itself. It need to further explore is that there are no binding sites in the promoters of gene α-SMA, COLIα2 and TGF-β for UTE1 by bioinformatics analysis, however, the treatment of UTE1 decoy ODN still has great impact at the expression level of these genes. In human fibroblasts of urethral scar as previously mentioned [29], overexpression of TIMP1 significantly enhanced expression of TGF-β, α-SMA, collagen I and induced transformation of fibroblasts into myofibroblasts, while inhibition of TIMP-1 by lentiviruses carrying a transgene or a short hairpin small interfering RNA for TIMP-1 significantly decreased TGF-β, α-SMA and collagen I levels. In our study, the expression of TIMP1 can be down-regulated by UTE1 Decoy ODN. It is a possible reason that the declined endogenous TIMP1 by UTE1 Decoy ODN leads to the down-regulated activity of the luciferase reporter gene of TGF-β, α-SMA and collagenIα2. Of course, it is worth further confirming mechanism in the future.

Recent studies have shown that TIMP1 plays an important role in colorectal cancer progression [33] and the expression of TIMP1 in HCC tissues is in consistent with cancer cell proliferation and advanced TNM stage [34]. Popov found that apoptosis of activated cholangiocytes and their subsequent macrophage mediated clearance were in favor of fibrosis reversal [35]. Host-derived TIMP1 can promote liver metastasis by inducing hepatocyte growth factor signaling which is conducive to liver proliferation [36]. SP1 plays an important regulatory role in cell proliferation [37] and SP1 Decoy ODN inhibits cell proliferation of hypertrophic scar fibroblasts [38]. SP1 Decoy ODN also can inhibit the proliferation of HSCs through down-regulating the expression of cyclin D1 and p27 [17]. Our research has shown that SP1 and UTE1 Decoy ODNs can inhibit HSC-T6 cell proliferation at 72 h but not 24 or 48 h. In a rabbit model, α-SMA-positive cell proliferation was inhibited through capturing both NFκB and E2F simultaneously by chimeric Decoy ODN [38]. Here, we have proved the combination of SP1 and UTE1 Decoy ODNs can restrain HSC-T6 cell proliferation at 72 h but not 24 or 48 h. Taken together, our study has declared that the combination of SP1 and UTE1 Decoy ODNs can inhibit the activation and proliferation of HSCs more effectively than one of Decoy ODNs through co-regulation of TIMP1 and TGF-β signal pathway but not the expression of TGF-β itself.

## Methods

### Synthesis of oligodeoxynucleotides (ODNs) and plasmid construction

The Decoy ODNs and scrambled (Scr) Decoy ODN (Table 1) were synthesized by Sangon Biotech (Shanghai, China). These Decoy ODNs were annealed overnight while the temperature decreased from 100 °C to 25 °C.

**Table 1:** The sequences of each Decoy ODN

Decoy SP1-F	5′-CTTGAACCCCGCCCCTCCT-3′
Decoy SP1-R	5′-AGGAGGGGCGGGGTTCAAG-3′
Decoy UTE1-F	5′-GGTAATGTGGTTTCCGATCC-3′
Decoy UTE1-R	5′-GGATCGGAAACCACATTACC-3′
Scramble-F	5′-GATCGGTACGGTACGAGC-3′
Scramble-R	5′-GCTCGTACCGTACCGATC-3′

Eukaryotic expression plasmid pGLuc-TRE-MiniTK was constructed when TGF-β responsive element (TRE) was cloned into the pGLuc-Mini-TK (BioLab, UK). Eukaryotic expression plasmids pGLuc-PSMA, pGLuc-P-COLΙα2 and pGLuc-P-TIMP1 were constructed when promoters of α-SMA (PSMA), COLΙα2 (P-COLΙα2) and TIMP1 (P-TIMP1) were also cloned into the pGLuc-Basic vector (N8082S, NEB, United States), respectively, for luciferase assays.

### HSC-T6 cell culture, transfection and luciferase reporter assays

HSC-T6 cells, an immortalized rat HSC line provided by the institute of liver disease at Shanghai University of Traditional Chinese Medicine, were cultured in high glucose DMEM (invitrogen) supplemented with 10 % newborn calf serum (NBCS). HSC-T6 cells were seeded in a 6-well-plate (Greiner, Germany) for western bolt assay or 24-well-plate (Greiner, Germany) for PCR or luciferase assays or 96-well-plate (Greiner, Germany) for MTT assay at 60 % confluence per well and cultivated in a humidified atmosphere containing 5 % CO2 for 24 h at 37 °C.

After plasmids were transfected into HSC-T6 cells using the Tubofect Transfection Reagent (Thermo) according to the manufacturer’s instructions for 24 h, respectively, cells were transfected with different Decoy ODNs(the concentration of SP1, UTE1 and Scr decoy ODN was 20 Nm/L, and the concentration of the decoy ODNs combination was also 20 Nm/L, containing 10 Nm/L of each single ODN) using the Mirus Transfection Reagent (Mirus Bio Corporation) for another 24 h. For luciferase assays, the supernatants were collected and the assays were performed using the Gaussia Luciferase Assay Kit (BioLux) according to the manufacturer’s instructions. The reactions were examined using a Fluorescence Detector (Brethold).

### Western blot analysis

Cells were collected for western blot assay after Decoy ODNs were transfected into HSC-T6 cells for 48 h. Then cells were lysed in lysis buffer (25 mmol/L Tris–HCl pH 7.5, 2.5 mmol/L EDTA, 137 mmol/L NaCl, 2.7 mmol/L KCl, 1 % sodium deoxycholic acid, 0.1 % SDS, 1 % TritonX-100, and 2 mmol/L PMSF) and protease inhibitor cocktail for 30 min at 4 °C. The cell lysates were clarified by centrifugation at 12,000 rpm for 20 min at 4 °C, and the supernatants were collected. The protein concentrations were measured using a BCA Protein Assay kit (Thermo). An equal amount of protein from each sample was separated by sodium dodecyl sulfate–polyacrylamide gel electrophoresis (SDS-PAGE) and transferred to a polyvinylidene difluoride membrane. The membrane was incubated with a mouse monoclonal anti-α-SMA antibody (1:1000 dilution) (Sigma), a rabbit polyclonal anti-TGF-β antibody (1:1000 dilution) (SANTA CRUZ), a goat monoclonal anti-(TIMP1, MMP2, COLΙα2) antibody (1:1000 dilution) (SANTA CRUZ) and a mouse monoclonal anti-β-actin antibody (1:3000 dilution) (Sigma) overnight at 4 °C. This primary antibody incubation was followed by incubation with HRP-conjugated anti-mouse (1:3000 dilution), anti-rabbit (1:3000 dilution) or anti-goat (1:8000 dilution) antibody as the secondary antibody for 1 h at room temperature. These membranes were developed using Immobilon Western Detection Reagents (Millipore) according to the manufacturer’s instructions. The chemiluminescence on the membrane was detected using the VersaDoc system (Bio-Rad). Densitometric analyses of the band intensities were performed using ImageJ software (version 1.38×; National Institutes of Health).

### Cell proliferation assay

After HSC-T6 cells were dealt with 20 nM of Decoy ODNs in triplicate for 24, 48 and 72 h, 50 μl MTT solution (250 μg/ml in DMEM) was added into each well and the cells were incubated at 37 °C for 4 h. When the plates were centrifuged at 380×*g* for 10 min, the supernatant medium was removed and then 200 μl of dimethylsulfoxide was added to each well for another 20 min. The absorbance (A) of each well at 490 nm was recorded. The cell survival rate was calculated according to the following formula: Cell survival rate (%) = A_Decoy_/A_control_ × 100.

### Statistical analysis

Data are presented as mean ± standard error (SE) of several experiments. Difference between two groups was analyzed by a two-tailed Student’s t test, and difference between three or more groups was analyzed by one-way ANOVA multiple comparisons. p < 0.05 was considered statistically significant.
